# Enhanced Biochemical and Structural Defense in PGPR-Inoculated Sweet Basil Under Aphid Herbivory

**DOI:** 10.3390/plants15010015

**Published:** 2025-12-20

**Authors:** Jimena Sofía Palermo, Tamara Belén Palermo, Lorena del Rosario Cappellari, Gerd Ulrich Balcke, Erika Banchio

**Affiliations:** 1 INBIAS Instituto de Biotecnología Ambiental y Salud (CONICET-Universidad Nacional de Río Cuarto), Campus Universitario, Río Cuarto 5800, Argentina; jpalermo@exa.unrc.edu.ar (J.S.P.); tpalermo@ayv.unrc.edu.ar (T.B.P.); lcappellari@exa.unrc.edu.ar (L.d.R.C.); 2 Leibniz Institute of Plant Biochemistry, Department of Cell and Metabolic Biology, Weinberg 3, 06120 Halle (Saale), Germany; gerd.balcke@ipb-halle.de

**Keywords:** *Ocimum basilicum*, *Bacillus amyloliquefaciens* GB03, *Acyrthosiphon pisum*, plant growth-promoting rhizobacteria, induced systemic resistance, volatile organic compounds, essential oil, total phenolic content, glandular trichome density

## Abstract

Plants are naturally exposed to various biotic stresses, including pathogen attacks and insect herbivory, which activate distinct signaling pathways as part of their defense responses. Inoculation with beneficial microorganisms, such as plant growth-promoting rhizobacteria (PGPR), can trigger induced systemic resistance (ISR) in plants, a defense response that resembles the one activated by herbivore attack in terms of signaling pathways and physiological effects. However, these interactions have typically been studied independently, limiting our understanding of their combined effects. In this study, we examined the effects of aphid (*Acyrthosiphon pisum*) herbivory on *Ocimum basilicum* plants and assessed how these responses are modulated when the plants are inoculated with the PGPR strain *Bacillus amyloliquefaciens* GB03, with a particular focus on biochemical and structural defense mechanisms. Aphid herbivory significantly increased total essential oil (EO) content and volatile organic compound (VOC) emission and induced a greater density of glandular trichomes while also modifying the phytohormone profile. In contrast, total phenolic content remained unchanged. When aphid herbivory occurred on GB03-inoculated plants, the effects on defense-related parameters became more pronounced. EO and eugenol contents were further increased compared with inoculated controls, jasmonates remained comparable to levels induced by either factor alone, and SA levels nearly doubled relative to aphid-infested plants. Feeding assays revealed that aphids preferred inoculated plants over controls, a response that may be explained by the increased emission of eugenol in inoculated basil. These results demonstrate that GB03 inoculation modifies several defenses-related responses in *O. basilicum* upon aphid herbivory, including by hormonal signaling, specialized metabolites accumulation, and structural barriers such as glandular trichomes. These findings suggest that PGPR may contribute to modulating plant responses to herbivory under certain conditions, highlighting their context-dependent influence within plant–microbe–insect interactions.

## 1. Introduction

Sweet basil (*Ocimum basilicum* L.) is an annual aromatic plant extensively cultivated for its culinary, medicinal, and aromatic properties. It is considered one of the most economically important species among aromatic crops. It is highly valued for its diverse profile of secondary metabolites (SMs), including essential oils (EOs) and phenolic compounds, which not only exhibit strong antioxidant activity and associated health benefits but also hold considerable potential as functional ingredients and biologically active compounds [1,2,3].

Like many crops, sweet basil is frequently exposed to biotic stressors, including insect herbivory, which can reduce plant performance and alter the accumulation of bioactive compounds. Aphids rank among the most significant agricultural pests affecting plants globally, accounting for 26% of the 45 key crop pests impacting major food crops in temperate regions [4]. They are capable of rapidly increasing their numbers due to their high reproductive rates (parthenogenesis), brief generation intervals, and the large percentage of viviparous females present within the population. Furthermore, fluctuations in population size over short periods and lack of adequate host nutrition lead to the emergence of winged forms that can be carried long distances by wind currents [5].

Aphids are specialized phloem-feeding herbivores that use an elongated mouthpart, the stylet, to probe plant tissues and reach the sieve elements. During feeding, they secrete saliva containing effector molecules that manipulate plant defense responses and prevent sieve-tube occlusion [6,7]. As a result, aphids can feed continuously while suppressing host defenses. They are potent pests on almost all crops, causing significant yield losses and economic damage worldwide [8]. Compared with chewing insects such as lepidopteran larvae, which cause extensive tissue damage, aphids impose a more subtle but persistent stress that involves the injection of salivary effectors and the modulation of plant physiology. These differences in feeding strategies are highly relevant because they may result in distinct patterns of hormone signaling and secondary metabolism [8,9,10]. Despite the economic and ecological importance of aromatic plants, very few studies have investigated how aphid herbivory influences the accumulation of secondary metabolites.

Plants have evolved sophisticated defense strategies to mitigate the impact of insect herbivory. These include the activation of phenolic metabolism, the production of EOs, and the emission of volatile organic compounds (VOCs) that may function either as repellents or as attractants of natural enemies. Structural defenses, such as glandular trichomes serving as reservoirs of EOs, also play a critical role by acting as a physical and chemical barrier against certain herbivores, particularly aphids [11].

Alongside these herbivore-induced responses, the deployment of plant defenses is orchestrated by phytohormonal signaling networks, with jasmonic acid (JA) and salicylic acid (SA) serving as central regulators of local and systemic resistance [12,13]. Herbivore feeding can strongly influence terpenoid and phenylpropanoid biosynthesis, as the accumulation of specialized metabolites under stress is frequently mediated by JA-dependent transcriptional activation of biosynthetic genes [14,15]. Beneficial microorganisms such as PGPR can also enhance plant resistance through induced systemic resistance (ISR), a response primarily mediated by JA and ethylene signaling [16,17,18,19,20]. PGPR inoculation has been reported to modulate hormonal balance, increase the production of defensive metabolites, and improve tolerance to pests and pathogens [16,17]. Notably, ISR and herbivore-induced pathways exhibit considerable mechanistic overlap, particularly in their reliance on JA-associated signaling, suggesting that PGPR may influence the plant’s response to herbivory through shared regulatory networks.

In the presence of chewing herbivory, PGPR-inoculated plants exhibited enhanced defense responses [19,20], supporting the idea that PGPR prime plants for a stronger defensive reaction [19,20,21,22]. Altogether, this evidence underscores the importance of considering both microbial partners and herbivore feeding guilds when analyzing plant defense regulation. Despite increasing interest in plant–PGPR–insect interactions, the impact of PGPR on sap-feeding herbivores remains poorly understood.

Nonetheless, it remains unclear the response to sap-feeding insects such as aphids, which impose less conspicuous but continuous stress. Evidence suggests that aphid herbivory often involves stronger activation of SA-related pathways, although JA signaling can also be engaged depending on the plant–insect system [8,17,21,22]. Thus, the present study investigates the effects of *Acyrthosiphon pisum* herbivory on the biochemical, structural, and phytohormonal responses of *Ocimum basilicum*, and evaluates how these responses are modulated in plants previously inoculated with *Bacillus amyloliquefaciens* GB03.

## 2. Results

### 2.1. Secondary Metabolites

#### 2.1.1. EO Yield and Changes in Composition

**Figure 1:**
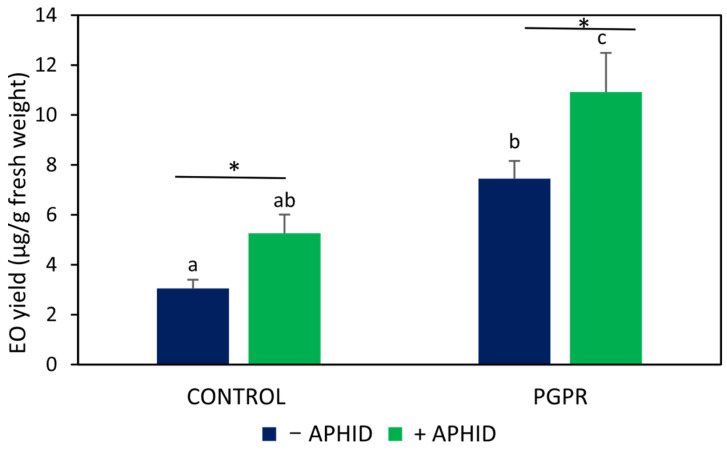
EO yields in *O. basilicum* plants inoculated with the PGPR strain *B. amyloliquefaciens* GB03 and/or subjected to herbivory by *A. pisum*. Values are means ± standard error (SE) (*n* = 11). Different letters above bars indicate significant differences according to Fisher’s LSD test (*p* < 0.05). Asterisks denote significant differences between aphid-infested and non-infested plants within the same inoculation treatment, according to Student’s *t*-test (*p* < 0.05).

Chromatographic analysis revealed that inoculation with *B. amyloliquefaciens* GB03 increased EO yield by approximately 2.5-fold compared with the uninoculated control (*F* = 12.77, *p* < 0.05; Figure 1). Within uninoculated plants, EO yield was significantly higher in aphid-infested plants, showing an increase of about 70% compared with non-infested controls (*t* = 2.69, *p* < 0.05). A similar pattern was observed in PGPR-inoculated plants, where aphid herbivory further increased EO yield by approximately 50% compared with inoculated, non-infested plants (*t* = 2.17, *p* < 0.05). The highest EO yield was recorded in PGPR-inoculated plants subjected to aphid herbivory. However, the inoculation × herbivory interaction was not statistically significant (*F* = 0.65, *p* > 0.5).

Regarding the main components of the EOs, inoculation with GB03 in the absence of aphids increased linalool, terpineol, and eugenol contents compared with uninoculated controls, with eugenol showing an approximately 2.7 fold increase (*F*_linalool_ = 5.88, *F*_terpineoll_ = 9.67, *F*_eugenol_ = 14.64, *p* < 0.05; Table 1). Within uninoculated plants, aphid infestation significantly increased linalool, terpineol and eugenol contents by 91%, 90% and 173%, respectively, compared with non-infested controls (*t*_linalool_ (2.30), *t*_terpineol_ (4.60), *t*_eugenol_ (3.28), *p* < 0.05). In PGPR-inoculated plants, aphid herbivory further increased terpineol (83%), and eugenol (71%) contents relative to inoculated plants without aphids (*t*_terpineol_ (2.17), *t*_eugenol_ (2.44), *p* < 0.05), whereas the increase in cineole and linalool was not statistically significant.

#### 2.1.2. Emission of Plant VOCs and Major Components

**Figure 2:**
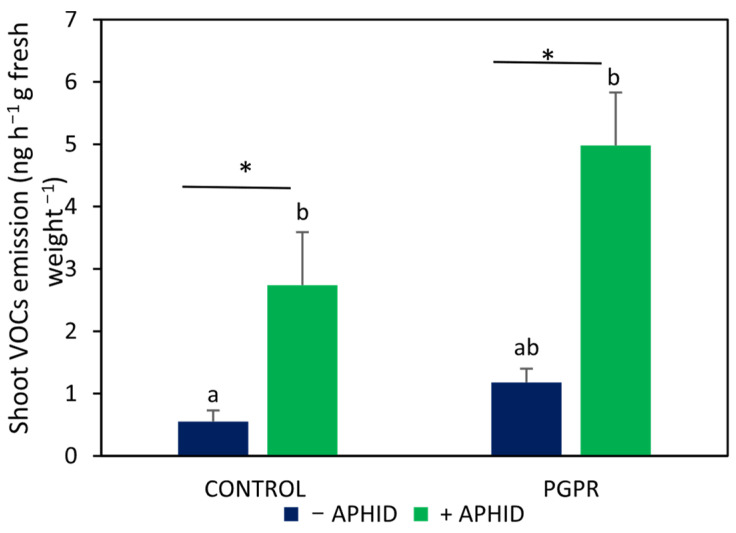
VOC emission in *O. basilicum* plants inoculated with the PGPR strain *B. amyloliquefaciens* GB03 and/or subjected to herbivory by *A. pisum*. Values are means ± standard error (SE) (*n* = 10). Different letters above bars indicate significant differences according to Fisher’s LSD test (*p* < 0.05). Asterisks denote significant differences between aphid-infested and non-infested plants within the same inoculation treatment, according to Student’s *t*-test (*p* < 0.05).

The plant VOC emission was similar between PGPR-inoculated plants and uninoculated controls (*F* = 9.91, *p* > 0.05) (Figure 2). In uninoculated plants, aphid infestation increased VOC emission by approximately 5-fold compared with non-infested controls (*t* = 3.09, *p* < 0.05). In PGPR-inoculated plants, aphid herbivory caused an approximately 4-fold increase relative to inoculated plants without aphids (*t* = 4.34, *p* < 0.05), resulting in the highest VOC levels detected in this study. These results indicate additive effects of PGPR inoculation and aphid herbivory on VOC emission, with no evidence of interaction between the two factors (*F* = 1.34, *p* > 0.5).

Regarding the major components of the volatile profile, in the absence of aphids, VOC levels were similar between PGPR-inoculated plants and uninoculated controls, except for eugenol, which increased significantly in inoculated plants (*F*_eugenol_ = 5.18, *p* < 0.05; Table 2). In uninoculated plants, aphid infestation did not significantly alter the VOC profile compared with non-infested controls, except for eugenol, which was approximately 8-fold higher in aphid-infested plants (*t*_eugenol_ = 2.30, *p* < 0.05; Table 2). In PGPR-inoculated plants, aphid herbivory caused marked increases in all major VOCs, with cineole increasing approximately 2.7-fold, linalool 2.8-fold, terpineol 5.4-fold, and eugenol 5.2-fold relative to inoculated plants without aphids (*t*_cineole_ = 2.60, *t*_linalool_= 2.70, *t*_terpineole_ = 4.68, *t*_eugenol_ = 5.32, *p* < 0.05). The highest concentrations of each major VOC were recorded in PGPR-inoculated plants subjected to aphid herbivory. However, no significant interaction between inoculation and herbivory was detected (Supplementary Table S1, *p* > 0.05).

#### 2.1.3. TPC and PAL Activity

**Figure 3:**
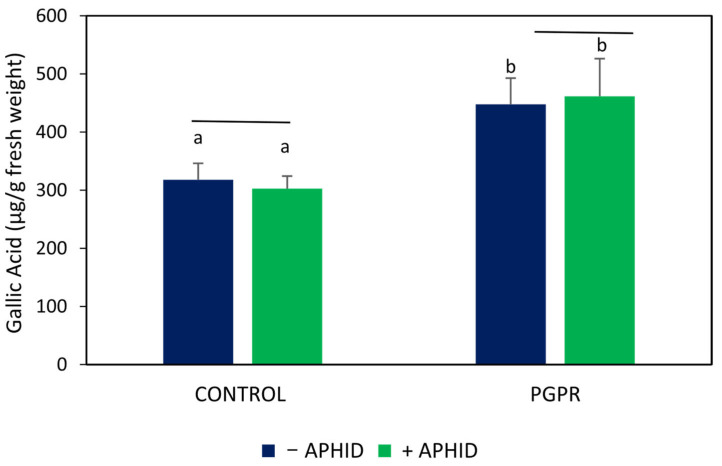
Total phenolic compounds in *O. basilicum* plants inoculated with the PGPR strain *B. amyloliquefaciens* GB03 and/or subjected to herbivory by *A. pisum*. Values are means ± standard error (SE) (*n* = 11). Different letters above bars indicate significant differences according to Fisher’s LSD test (*p* < 0.05).

The accumulation of phenolic compounds was higher in PGPR-inoculated plants than in uninoculated controls, with an increase of approximately 41% (*F* = 3.55, *p* < 0.05; Figure 3). Within uninoculated plants, aphid infestation did not significantly alter total phenolic content (*t* = 0.44, *p* > 0.05). Similarly, in PGPR-inoculated plants, aphid herbivory produced only a slight, non-significant increase compared with inoculated plants without aphids (*t* = 0.18, *p* > 0.05). The highest phenolic content values were recorded in PGPR-inoculated plants, regardless of the presence of aphids. No evidence of interaction between inoculation and herbivory was detected (*F* = 0.11, *p* > 0.05).

PAL activity was higher in PGPR-inoculated plants than in uninoculated controls, with an increase of approximately 82% in the absence of aphids (*F* = 3.32, *p* < 0.05; Table 3). Within uninoculated plants, aphid infestation increased PAL activity by about 65% compared with non-infested controls (*t* = 2.59, *p* < 0.05). In PGPR-inoculated plants, aphid herbivory did not significantly alter PAL activity relative to inoculated plants without aphids (*t* = 0.11, *p* > 0.05). The highest PAL activity values were recorded in PGPR-inoculated plants, regardless of the presence of aphids. No significant interaction between inoculation and herbivory was detected (*F* = 1.99, *p* > 0.05).

### 2.2. Gene Expression of Key Enzymes of EO Main Components

**Figure 4:**
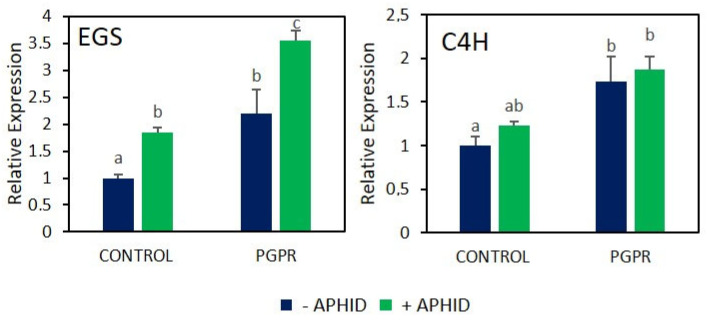
Relative expression of *C4H* and *EGS* genes in *O. basilicum* plants inoculated with the PGPR strain *B. amyloliquefaciens* GB03 and/or subjected to herbivory by *A. pisum*. Values are means ± standard error (SE) (*n* = 3). Different letters indicate significant differences according to Fisher’s LSD test (*p* < 0.05).

The different treatments applied—PGPR inoculation, aphid infestation, and their combination—significantly affected the production of EOs in *O. basilicum*. To gain deeper insight into how these factors influence the biosynthesis of key constituents, the relative expression of the genes cinnamate-4-hydroxylase (*C4H*) and eugenol synthase (*EGS*), both involved in EO metabolism, was analyzed by qPCR (Figure 4). The expression of *C4H* varied depending on the treatment applied. Inoculation with PGPR significantly upregulated *C4H* expression, showing an approximately 2-fold increase compared with uninoculated controls (*F* = 4.10, *p* < 0.05). Aphid infestation in uninoculated plants also induced a significant increase in *C4H* expression relative to control plants (*t* = 5.84, *p* < 0.05). However, when both factors were combined (PGPR + aphids), expression levels were similar to those observed in inoculated plants without herbivory (*t* = 0.31, *p* > 0.05). Regarding *EGS*, inoculation with PGPR significantly increased *EGS* expression compared with uninoculated controls (*F* = 22.10, *p* < 0.05). In uninoculated plants, aphid feeding also led to an increase in *EGS* transcript levels relative to undamaged controls (*t* = 13.49, *p* < 0.05). Moreover, when aphid herbivory occurred on PGPR-inoculated plants, *EGS* expression increased even further compared with inoculated plants without aphids, representing the highest levels observed among all treatments (*t* = 4.51, *p* < 0.05).

### 2.3. Endogenous Phytohormones

**Figure 5:**
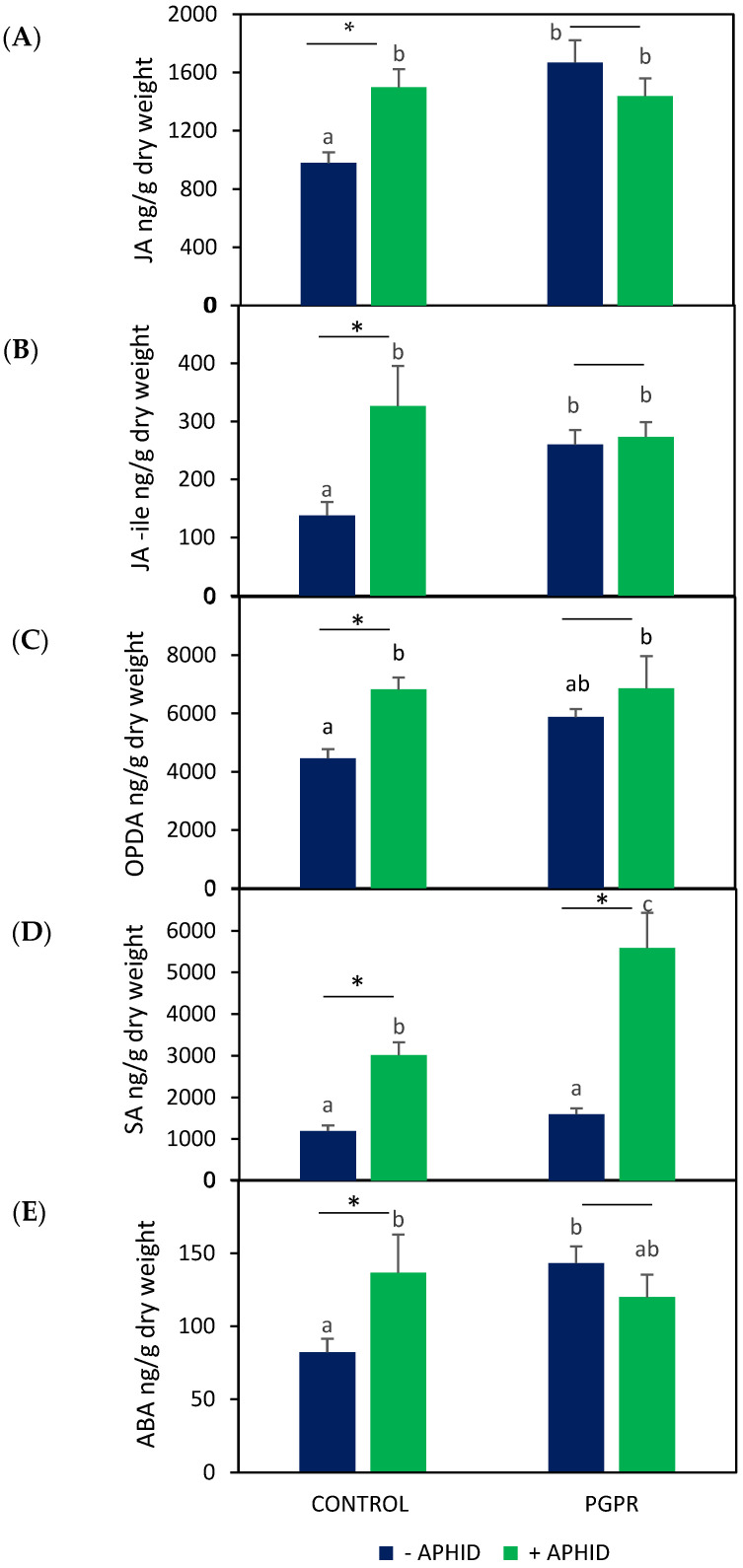
Endogenous phytohormone content, (**A**) JA, (**B**) JA-ile, (**C**) OPDA, (**D**) SA, (**E**) ABA, in *O. basilicum* plants inoculated with *B. amyloliquefaciens* GB03 and/herbivory by *A. pisum*. Values are means ± standard error (SE) (*n* = 9). Different letters indicate significant differences according to Fisher’s LSD test (*p* < 0.05). Asterisks denote significant differences between aphid-infested and non-infested plants within the same inoculation treatment, according to Student’s *t*-test (*p* < 0.05).

To further understand the regulation of defense responses underlying the observed changes in EO, plant VOCs emission, and TPC, we quantified the endogenous levels of JA, jasmonoyl-isoleucine (JA-Ile), 12-oxo-phytodienoic acid (OPDA), SA, and abscisic acid (ABA). The jasmonates evaluated—JA, JA-Ile, and OPDA—showed similar response patterns across treatments (Figure 5A–C). Inoculation with PGPR increased the levels of all three phytohormones by approximately 1.4- to 1.9-fold compared with uninoculated controls (*F*_JA_ = 5.83, *F*_JA-Ile_ = 3.87, *F*_OPDA_ = 3.20 *p* < 0.05). Aphid herbivory in non-inoculated plants also enhanced jasmonate accumulation, with JA and OPDA showing approximately 1.5-fold increases and JA-Ile increasing about 2.3-fold relative to control plants (*t*_JA_ = 3.61, *t*_JA-Ile_ = 2.59, *t*_OPDA_ = 4.55, *p* < 0.05). However, in inoculated plants subjected to aphid herbivory, jasmonate levels remained similar to those of inoculated plants without insects (*t*_JA_ = 1.16, *t*_JA-Ile_ = 0.37, *t*_OPDA_ = 0.86, *p* > 0.05).

SA levels also varied with the treatment applied (Figure 5D). Inoculation with PGPR alone did not produce significant changes compared with control plants (*F* = 17.83, *p* > 0.05). In contrast, aphid infestation in uninoculated plants led to a marked increase in SA content, reaching approximately 2.5-fold higher levels than in control plants (*t* = 5.34, *p* < 0.05). When aphid herbivory occurred on PGPR-inoculated plants, SA levels further increased, showing about a 3.5-fold rise relative to control plants (*t* = 4.69, *p* < 0.05).

ABA levels increased in PGPR-inoculated plants compared with control plants (*F* = 2.58, *p* < 0.05, Figure 5E). In uninoculated plants, aphid infestation also promoted higher ABA accumulation relative to control plants (*t* = 3.76, *p* < 0.05). However, in inoculated plants subjected to aphid herbivory, ABA levels remained similar to those of inoculated plants without insects (*t* = 1.21, *p* > 0.05).

### 2.4. Glandular Trichome Density

**Figure 6:**
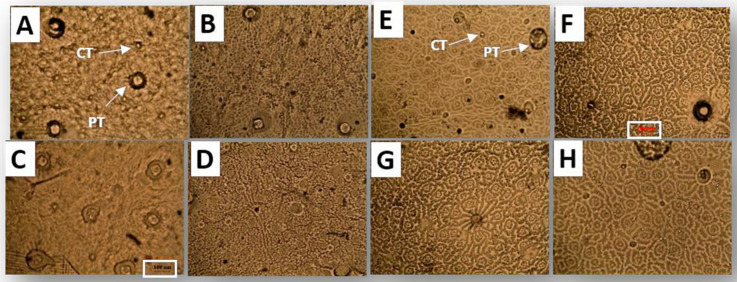
Microphotographs of the leaf surfaces of *O. basilicum* plants inoculated with *B. amyloliquefaciens* (GB03) and/or exposed to aphid feeding. (**A**) Control abaxial; (**B**) PGPR abaxial; (**C**) Aphid abaxial; (**D**) PGPR + Aphid abaxial; (**E**) Control adaxial; (**F**) PGPR adaxial; (**G**) Aphid adaxial; (**H**) PGPR + Aphid adaxial.

Glandular peltate and capitate trichomes, the structures responsible for synthesizing and storing monoterpene-rich EO, were present on both adaxial and abaxial leaf surfaces (Figure 6). On the abaxial surface, the density of peltate trichomes (PT) was higher in PGPR-inoculated plants than in uninoculated controls, with increases of approximately 40% in the absence of aphids (*F* = 3.99, *p* < 0.05; Table 5). Aphid infestation in uninoculated plants also increased PT density approximately 35% (*t* = 2.26, *p* < 0.05), whereas in PGPR-inoculated plants, aphid herbivory did not significantly alter the density relative to inoculated plants without aphids (*t* = 0.89, *p* > 0.05). A similar response pattern was observed on the adaxial leaf surface. The density of capitate trichomes (CT) on the abaxial surface remained similar among all treatments (*F* = 1.40, *p* > 0.05). On the adaxial surface, CT density did not differ between PGPR-inoculated and uninoculated plants under non-herbivory conditions. Aphid infestation significantly increased CT density in uninoculated plants compared with uninoculated, non-infested plants (*t* = 2.10, *p* < 0.05). Similarly, in inoculated plants, aphid herbivory resulted in higher CT density than in uninoculated, non-infested plants (*t* = 2.32, *p* < 0.05), with the highest values among all treatments.

### 2.5. Principal Component Analysis

**Figure 7:**
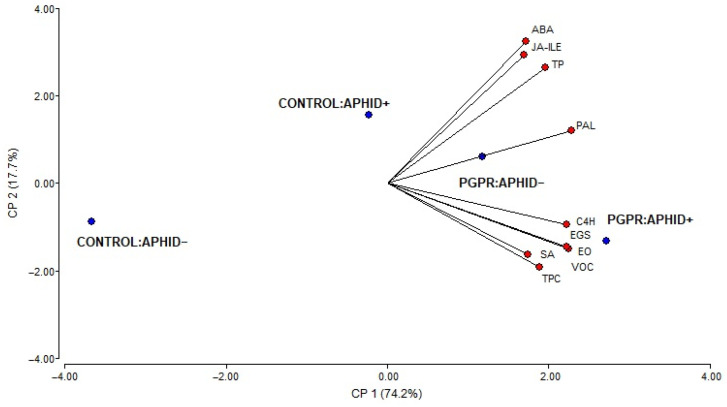
Principal component analysis of the physiological and biochemical responses of *O. basilicum* plants subjected to *A. pisum* herbivory and PGPR inoculation with *B. amyloliquefaciens* GB03 strain (CONTROL/APHID−, CONTROL/APHID+, PGPR/APHID−, PGPR/APHID+). Variables included EO, TPC, JA-Ile, SA, ABA, PAL, VOC, and Relative gene expression of *EGS* and *C4H*.

In order to relate the treatments, aphid herbivory, PGPR inoculation, and their combination, with the factors evaluated (EOs yield, TP density, TPC, PAL activity, *EGS* and *C4H* gene expresion and endogenous phytohormone levels, ABA, JA-ile, SA), a multivariate principal component analysis (PCA) was performed. In the PCA (Figure 7), for the insect herbivory and inoculation conditions, PC1 accounted for 74.2%, and PC2 for 17.7%, of the total variability in the data. Together, both axes explained 91.9% of the total variance and provided a cophenetic correlation coefficient of 0.991, indicating an excellent preservation of the original distance relationships in the reduced-dimensional space. The distribution of treatments along PC1 revealed a clear separation between inoculated and uninoculated plants, indicating that inoculation with PGPR was the main factor driving the variability in the data set. Variables associated with metabolic activation, such as *C4H* and *EGS* expression, EO and VOC emission, TP density, and SA, were positively correlated with inoculated treatments, particularly with the combined treatment (PGPR + aphids), which appeared on the right side of the plot. In contrast, uninoculated plants, especially those without aphids, were located on the negative side of PC1 and were associated with lower values of these variables.

PC2, which explained a smaller proportion of variance, partially differentiated plants according to aphid herbivory. However, this separation was less pronounced than that observed for inoculation. JA-Ile and ABA contributed most to the positive values of PC2, reflecting their higher levels in aphid-infested plants regardless of inoculation. Overall, the PCA highlights that PGPR inoculation exerted a stronger influence than aphid herbivory on the biochemical and structural defense traits of *O. basilicum*, while aphid feeding mainly affected hormonal variables associated with stress signaling.

### 2.6. Feeding Preference Assay

**Figure 8:**
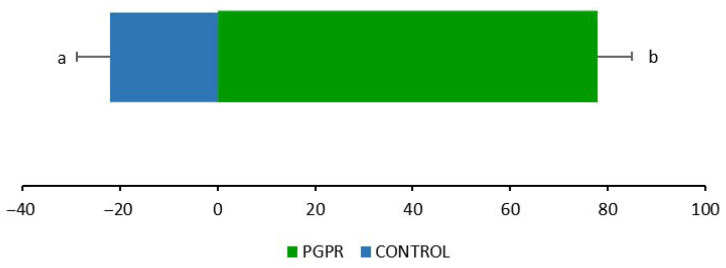
Feeding preference (%) of *A. pisum* between whole plants of *O. basilicum* plants inoculated or not with *B. amyloliquefaciens* GB03. Different letters indicate significant differences, as calculated by a two-tailed *t*-test (*p* < 0.05).

To evaluate whether rhizobacterial inoculation affected aphid host preference, a choice test was conducted comparing the proportion of aphids settled on *O. basilicum* plants with and without inoculation. Aphids exhibited a significantly higher preference for PGPR-inoculated plants compared with uninoculated controls (*t* = 4.37, *p* < 0.05). This corresponds to approximately 80% of aphids settling on inoculated plants versus 20% on controls (Figure 8), indicating that rhizobacterial inoculation markedly increased host acceptance by aphids.

## 3. Discussion

Our findings align with previous studies reporting increases in EO yield, eugenol content, TPC, and PAL activity following inoculation of *O. basilicum* with the PGPR *B. amyloliquefaciens* GB03, although the magnitude and consistency of these effects may vary across experimental conditions [18,19,20], accompanied by an increase in jasmonates (JA, JA-Ile, and OPDA). Similar stimulatory effects on secondary metabolism have been reported in other aromatic species [23,24,25,26,27], indicating the activation of signaling pathways associated with defense and secondary metabolism. Altogether, these metabolic adjustments are consistent with early defense-related metabolic activation but cannot be interpreted as ISR given the short herbivory window and the lack of a significant interaction between factors [28,29]. In line with this, studies on Mentha piperita detected increased plant VOC emissions after inoculation with GB03 or co-inoculation with *Pseudomonas fluorescens* SJ04 [25], and Banchio et al. [18] similarly reported that exposure of *O. basilicum* plants to bacterial VOCs released by GB03 under in vitro conditions enhanced VOC production. In contrast, in the present study, GB03-inoculated plants did not show a significant increase in VOC emission. This discrepancy may be attributed to differences in growth substrate and inoculation method, since plants in this work were grown in vermiculite and directly inoculated at the root, rather than being exposed to bacterial VOCs.

### 3.1. Effects of Aphid Herbivory on O. basilicum Defense Metabolism

Aphid feeding significantly affected several physiological and metabolic parameters in *O. basilicum* plants. The increase in total EO content and in the abundance of major compounds observed in this study may be interpreted as part of the plant’s defensive strategy. A similar pattern of enhanced EO accumulation and altered secondary metabolism was previously observed in *O. basilicum* plants damaged by *S. frugiperda* [19]. EOs are known to possess insecticidal properties, affecting the nervous system and other vital functions of insects, and many also act as repellents with contact toxicity, interfering with development and reproduction [30,31]. For instance, the EO from *O. campechianum* exhibited larvicidal activity against *Aedes aegypti*, increasing larval mortality after 24 h of exposure [32].

Eugenol, in particular, has been reported as a potent insecticide effective against a wide range of arthropod pests and also exhibits antimicrobial activity [33]. The observed increase in eugenol content in plants infested with aphids was linked to elevated activity of PAL and enhanced expression of the gene *C4H*. The biosynthesis of phenylpropanoids, particularly eugenol, begins with the deamination of the amino acid phenylalanine, a reaction catalyzed by PAL that generates cinnamate. This cinnamate is then hydroxylated to produce p-coumarate, facilitated by the action of cinnamate-4-hydroxylase. Ultimately, eugenol is synthesized through a series of reactions that convert coniferyl acetate, with the final step catalyzed by eugenol-synthase [34].

In relation to the VOCs emitted by *O. basilicum* plants damaged by aphids, a significant increase was detected. This finding has also been reported in other plant species infested by aphids, such as faba bean attacked by *A. pisum* [6], and tomato, where increased release of α-pinene and methyl salicylate was observed following infestation by *Myzus persicae* [35]. Similarly, Staudt et al. [36] documented enhanced VOC emissions in peach varieties infested with *M. persicae*. A comparable pattern was also observed in *O. basilicum* plants subjected to chewing herbivory by *S. frugiperda*, where a significant increase in total VOCs was detected [19].

Regarding eugenol emission, a clear increase was observed in aphid-damaged basil plants. This contrasts with the findings of Tozin et al. [37], who reported that in *Ocimum gratissimum* plants attacked by leaf-cutting ants, eugenol and terpineol contents decreased while cineole levels remained unchanged. These contrasting responses suggest that the type of herbivory and the feeding behavior of the insect can distinctly modulate the biosynthesis and release of specific volatile compounds.

The observed increase in VOC emission may represent a defensive strategy in *O. basilicum*. Herbivory-induced volatiles play a key ecological role by mediating tritrophic interactions, as they attract natural enemies of herbivores such as parasitoids and predators, thereby enhancing indirect plant defense [38,39,40,41].

Phenolic compounds showed no significant changes in total content in *O. basilicum* plants fed upon by *A. pisum*. In contrast, Rashid et al. [42] reported a strong increase in TPC in various peanut genotypes attacked by the chewing herbivore *Helicoverpa armigera* and the piercing-sucking insect *Aphis craccivora*. Gaur et al. [43] also found that *Populus tremula* plants damaged by the aphid *Chaitophorus* showed altered phenolic levels compared with undamaged controls. Moreover, Wu et al. [44] observed cultivar-dependent responses in *Medicago sativa* infested by three species of thrips (piercing-sucking herbivores): one cultivar exhibited a decrease relative to controls, whereas others showed an increase, suggesting that differences in phenolic accumulation may reflect specific physiological and biochemical adaptations. Consistent with the present results, our previous study showed that *O. basilicum* plants subjected to chewing herbivory by *S. frugiperda* did not exhibit significant changes in TPC [20].

The absence of a significant increase in TPC after *A. pisum* feeding is particularly notable, given that these compounds often play key roles in plant defense. Certain phenolic compounds can reduce plant digestibility and nutritional value, and in some cases negatively affect insect fitness [45]. The main phenolic compounds in basil are caffeic, sinapic, rosmarinic, and ferulic acids [46], which perform various ecological functions, including herbivore deterrence. Leaf extracts rich in rosmarinic acid from *O. gratissimum* have shown insecticidal activity against mosquito larvae [47]. Some aphids secrete polyphenol oxidases and peroxidases in their saliva, which detoxify phenolic compounds and may explain the limited TPC accumulation observed [48,49].

In the present study, an increase in PAL activity was observed in plants inoculated with GB03 or affected by aphids. Although PAL activity was generally accompanied by higher TPC, this correlation was not evident in insect-damaged plants. This apparent lack of correlation should be interpreted considering that eugenol, a representative phenylpropanoid-derived phenol, was not included in the TPC determination. Since total phenolics were quantified using the Folin–Ciocalteu method, which measures only water-soluble phenols, eugenol was excluded due to its low solubility in water resulting from its lipophilic nature. Therefore, the increased PAL activity observed in insect-damaged plants is likely reflected in the accumulation of total phenolics as well as in the enhanced biosynthesis of eugenol.

Hormonal signaling played a central role in the response of *O. basilicum* to aphid herbivory. Phloem-feeding insects typically induce SA-mediated defenses due to their limited tissue damage, often leading to the suppression of JA-dependent pathways [13,50]. Consistent with this, increased SA levels have been reported in *Lycium barbarum* infested by *Aphis gossypii*, where JA levels remained unchanged [51].

In contrast, our results revealed a simultaneous accumulation of SA and jasmonates (JA, JA-Ile, and OPDA) in aphid-damaged basil, suggesting that this species mounts a mixed defense response rather than the typical antagonistic pattern described for most aphid–plant systems. A similar co-activation of JA and SA pathways was also observed in cucumber attacked by *A. gossypii*, where upregulation of *OPR2* and *OPR11* enhanced JA biosynthesis and plant resistance [52]. In addition, ABA levels increased under aphid feeding, supporting its involvement in the coordination of stress responses rather than in susceptibility, as previously reported in soybean [53]. Altogether, these findings suggest that SA, JA, and ABA contribute independently yet concurrently to the activation of biochemical and structural defenses in basil, representing a flexible response strategy against phloem-feeding insects.

These divergent hormonal responses may be influenced by differences in aphid specialization. It is possible that generalist aphids, which have limited adaptation to a given host, occasionally trigger JA defenses detrimental to their own performance, whereas specialized species might have evolved strategies to mitigate or evade JA-mediated responses [8]. The extent of aphid specialization and the evolutionary history of plant–aphid interactions may therefore help predict the relative induction and impact of the JA and SA signaling pathways across different systems [8,54].

These results indicate that *A. pisum* activates both JA- and SA-dependent signaling pathways in basil. While aphids are phloem feeders typically associated with SA-mediated defense responses [8], the concurrent increase in JA suggests that basil mounts a mixed defense response integrating both hormonal pathways, possibly interpreted as concurrent activation rather than evidence of hormonal synergy or crosstalk. Such co-activation may reflect a dual strategy: SA-mediated systemic resistance aimed at phloem defense and JA-mediated production of deterrent metabolites and VOCs. In this context, JA regulates the biosynthesis of secondary metabolites through JA-dependent transcription factors (e.g., MYC2), which activate the expression of key genes encoding enzymes in the phenylpropanoid and terpenoid pathways, thereby promoting the production of deterrent compounds and herbivore-induced VOCs [55]. These responses likely originate from the perception of aphid-derived elicitors by the host plant, which triggers intracellular signaling cascades involving key phytohormones such as SA, JA, and ET [56,57].

While the role of ABA in abiotic stress responses and pathogen resistance is well established [58,59,60], its involvement in plant–insect interactions remains less clear. Early models proposed that ABA acts synergistically with JA to induce defense responses against herbivory [61], but more recent studies indicate that this relationship is highly dependent upon the specific plant-insect system [60,62]. For instance, Thaler & Bostock [58] found that ABA did not alter JA expression in tomato but contributed to reduced *Spodoptera exigua* growth, suggesting a defensive role independent of JA signaling. In aphid–plant systems, ABA responses have been mainly associated with susceptibility rather than tolerance. In soybean, aphid infestation induced ABA accumulation and JA-related transcripts in susceptible genotypes but not in resistant ones, where the lack of induction may result from aphid-mediated suppression of defense signaling [53].

The increases in phytohormone concentrations observed in this study correlate with the enhanced accumulation of EOs and volatile compounds in basil plants damaged by aphids. The rise in these secondary metabolites upon stress exposure is regulated by interconnected hormonal signaling pathways involving JA/ET and SA, which coordinate the synthesis of terpenes, flavonoids, and other metabolites that play crucial roles in plant defense against a wide range of pathogens and herbivores [63,64]. This integrated hormonal regulation underscores the capacity of *O. basilicum* to mount a complex and flexible defense network in response to phloem-feeding insects.

In relation to structural traits, glandular trichomes play a key role in plant chemical defense [63]. In our study, the increases in EO content observed in aphid-attacked plants were consistent with the higher density of PT recorded on the abaxial leaf surface of basil. Similar results were observed in basil plants subjected to herbivory by *S. frugiperda* [19]. This observation is consistent with expectations, since the enzymatic machinery required for EO biosynthesis is localized within PT [37]. It is also important to highlight that leaf trichomes can interfere with insect oviposition and feeding, thus constituting a structural and chemical protection together with the production of secondary metabolites [65,66]. Although the present study did not assess aphid performance or feeding damage, and therefore no functional defensive effect can be inferred, the observed increase in trichome density should be interpreted as a structural response rather than a demonstrated defensive outcome. For aphids, once they are attracted to a host plant by phytochemicals [67], the first leaf characteristic influencing their behavior is trichome presence. Regardless of their structure, trichome density substantially affects the success of aphid infestations [68]. For example, in the wild tomato species *Lycopersicon pennellii*, aphid establishment is reduced due to the presence of a high density of both simple and glandular trichomes [6]. In contrast with our results, where we observed an increase in PT density on both abaxial and adaxial leaf surfaces of plants affected by aphids, Tozin et al. [37] reported an increase in trichomes only on the adaxial surface of *O. gratissimum* leaves attacked by leaf-cutting ants. Such differences highlight that trichome responses can vary among species and herbivore systems, and some plants may show no induction of trichomes following herbivory.

In behavioral assays, adult *A. pisum* individuals showed a clear preference for GB03-inoculated plants compared with controls. This response may be associated with alterations in the volatile profile of inoculated basil, particularly the marked increase in eugenol emission, which was approximately twice that of control plants. Although total VOC release remained similar, the enrichment in eugenol suggests that this compound may act as an attractant, contributing to the observed host-selection behavior. The influence of other minor volatiles not quantified here cannot be ruled out. Moreover, estragole—another major constituent of basil essential oil—has been reported as a potential attractant of the winged melon aphid *Aphis gossypii* [69], further supporting the possibility that multiple components of the basil volatile blend may contribute to aphid attraction.

This apparent contradiction, enhanced biochemical defense markers together with increased aphid attraction, highlights the multifunctional roles of plant volatiles. For instance, although eugenol can participate in defense in some systems [33], it is also known to attract certain herbivores [70]. A similar pattern has been described in other plant–insect interactions, such as the *Ips typographus*–spruce system, where specific volatiles (e.g., verbenol) are strongly upregulated during the early stages of attack and facilitate host localization [71].

These observations illustrate that increases in defense-related metabolites do not necessarily result in reduced herbivore attraction. Many defense-associated compounds, including VOCs, have multifunctional ecological roles and can operate as host-location cues for aphids depending on their sensory preferences and metabolic specialization. Moreover, because our experiment captured early responses (30 h of feeding), biochemical activation can precede ecological outcomes such as deterrence or reduced host preference. Thus, the coexistence of enhanced defense markers and greater aphid attraction in GB03-treated plants is not contradictory but reflects the complexity of volatile-mediated interactions during the initial stages of herbivore attack.

Thus, while PGPR inoculation enhanced several defense-related biochemical traits, this does not necessarily result in reduced herbivore preference, particularly at early response stages. Increases in VOC emission should not be interpreted as inherently defensive, as some volatiles can operate simultaneously as attractants. Future studies integrating a more comprehensive VOC profiling and behavioral assays are needed to disentangle the relative contributions of individual compounds, such as eugenol, estragole, and other minor volatiles, to aphid host selection in PGPR-inoculated basil.

### 3.2. Herbivory Responses in GB03-Inoculated Plants

When *O. basilicum* plants inoculated with *B. amyloliquefaciens* GB03 were exposed to aphid herbivory, the overall biochemical response differed from that of the individual treatments. The combined stress condition resulted in the highest EO content and VOC emission recorded, indicating an additive effect of PGPR inoculation and aphid feeding on secondary metabolism. PAL activity also increased relative to aphid-infested plants, reaching values comparable to those observed in inoculated controls may be associated with higher PAL activity in inoculated plants. These results indicate that the combined treatment produced additive modifications in both biochemical and structural traits, suggesting that inoculation and herbivory contribute independently to the plant response. Since no statistically meaningful cross-effect between factors was detected in the analysis, these patterns cannot be interpreted as evidence of a true primed state [72,73]. The responses documented here correspond to early defense activation triggered during the first hours of aphid feeding and therefore should not be interpreted as long-term induced resistance.

At the hormonal level, the accumulation patterns of jasmonates (JA, JA-Ile, and OPDA) and ABA in the combined treatment remained similar to those of aphid-infested or PGPR-inoculated plants alone, suggesting a convergence of these signaling routes under dual stress. The significant SA induction, together with unchanged JA levels, indicates that GB03 inoculation was associated with higher SA levels under aphid feeding, consistent with defense patterns typically associated with phloem-feeding herbivores. Similar interactions were described by Pineda et al. [74], who reported that *P. simiae* WCS417r enhanced systemic resistance to aphids in *Arabidopsis thaliana* through the joint activation of SA- and JA-regulated defenses, while modulating ABA-associated responses.

The increases in JA and SA levels detected under the combined treatments (aphid herbivory plus PGPR inoculation) compared with control plants are consistent with the observations of Serteyn et al. [75], who reported higher JA and SA contents in faba bean plants inoculated with *B. amyloliquefaciens* and subsequently infested with *A. pisum*. Similarly, Kousar et al. [76] found increased SA levels in plants inoculated with *Bacillus endophyticus* or *Pseudomonas aeruginosa* and damaged by *Spodoptera litura*, compared with control, inoculated-only, or herbivory-only treatments. Following the same trend, Zebelo et al. [77] observed increased JA levels in cotton plants inoculated with PGPR mixtures and infested with *S. exigua* relative to controls. Likewise, Shavit et al. [78] reported that ISR triggered in PGPR-treated plants involves JA and ET signaling, either independently or through crosstalk with SA-dependent pathways, ultimately shaping insect–host interactions. overall defensive response was enhanced. Collectively, these studies show that PGPR can influence plant hormonal signaling under herbivory. In our study, however, the short exposure to aphids reflects early responses and does not allow inference of ISR or priming.

In previous studies where *O. basilicum* plants inoculated with *B. amyloliquefaciens* GB03 were subjected to chewing herbivory by *S. frugiperda* [19], similar outcomes to those observed in the present work were reported, with significant increases in EO content and VOC emission. However, the hormonal profiles differed markedly between both systems, showing in our study a clear predominance of SA over jasmonates. This divergence likely reflects the phloem-feeding behavior of *A. pisum* and underscores the specificity of PGPR-mediated defense modulation, which depends on the herbivore’s feeding strategy and the nature of the tripartite plant–microbe–insect interaction.

However, the influence of PGPR on herbivorous insects is not uniform. Previous research has shown neutral [79,80], negative [81,82], and even positive effects [83,84,85] on aphid performance. These contrasting outcomes are shaped by the specific combination of host plant, bacterial strain, and aphid species, particularly by the degree of aphid specialization [86]. For instance, *Pseudomonas fluorescens* WCS417r enhanced the performance of the generalist *Myzus persicae* but not that of the specialist *Brevicoryne brassicae* [74], whereas *B. amyloliquefaciens* FZB42 modified the life-history traits of *B. brassicae* on *Brassica oleracea* [82]. Likewise, Serteyn et al. [75] reported a reduction in the fecundity of the moderately specialized *A. pisum* on *Vicia faba* inoculated with PGPR, illustrating how rhizobacteria may differentially influence aphid fitness depending on host adaptation and specificity. Similar patterns were observed in *Capsicum annuum*, where PGPR inoculation with a commercial BioYield formulation did not suppress *M. persicae* infestations or enhance natural enemy abundance but did improve fruit yield and quality during specific harvests [80]. This suggests that rhizobacterial treatments can confer physiological benefits to the host plant without directly affecting aphid populations. In another system, Fahimi et al. [87] demonstrated that among several PGPR strains tested on cucumber, only strain PF169 significantly reduced the population growth rate of *Aphis gossypii*, indicating that strain-specific effects may determine the success of microbial applications in pest management programs. Furthermore, Nasab et al. [88] showed that canola plants inoculated with PGPR—alone or in combination with humic acid—displayed enhanced resistance to *B. brassicae*, manifested as reduced longevity, fecundity, and intrinsic rate of increase in the aphids. This induced resistance was associated with elevated levels of total phenolics, flavonoids, and glucosinolates, compounds central to both antibiosis and plant defense signaling.

## 4. Materials and Methods

### 4.1. Bacterial Strains, Culture Conditions and Media

The PGPR strain utilized in this study was *Bacillus amyloliquefaciens* GB03. It was cultured on LB medium and preserved in nutrient broth with 15% glycerol at −80 °C for long-term storage. For experimental purposes, the bacteria were cultured on LB nutrientagar. Single colonies were then transferred to 100 mL flasks containing the LB medium and grown aerobically on a rotating shaker (150 rpm) for 24 h at 28 °C. The resulting bacterial suspension was diluted in sterile saline solution (0.9% sodium chloride, NaCl) to achieve a final concentration of 10^8^ colony-forming units (CFU) per milliliter. Subsequently, 1 mL of this suspension was applied around the base stem of the plants [18]. It should be noted that GB03 colonization was not directly quantified in this experiment. However, this inoculation procedure has been widely validated in previous studies, including work on basil using the same strain and protocol; Banchio et al. [18], demonstrating reliable root association and physiological activity of GB03 under comparable conditions.

### 4.2. Seed Sterilization and Plant Cultivation

Seeds of *Ocimum basilicum* L. var. Genovese (Florensa Argentina S.A., Córdoba, Argentina) were surface sterilized by soaking for 2 min in 70% (*v*/*v*) ethanol and for 20 min in 1% (*v*/*v*) sodium hypochlorite. After this, they were thoroughly rinsed 4 times with sterile distilled water and placed in plastic pots filled with sterilized vermiculite. Following a 15-day period, the plantlets were transplanted into larger plastic pots (12 cm × 22 cm) filled with sterilized vermiculite [19]. They were grown in a growth chamber under controlled conditions of light (16/8 h light/dark cycle), temperature (22 ± 2 °C), and relative humidity (~70%) and watered every week with 20 mL of Hoagland solution per pot. After seven days, the plants were inoculated with 1000 µL of bacterial suspension or with saline solution in the case of control plants. The experiments were conducted three times (10 pots per treatment, one plant per pot) and arranged randomly in the growth chamber.

Each plant (one per pot) was considered an independent biological replicate. The experiment was repeated three times under identical environmental conditions, and data from the three experimental runs were pooled after confirming the absence of run effects or run × treatment interactions. Therefore, statistical analyses were performed on plant-level data pooled across runs.

### 4.3. Aphid Infestation and Experimental Design

Adult *Acyrthosiphon pisum* individuals were collected from *Medicago sativa* in the field, and these collected adults were the ones used for the bioassays. After collection, aphids were kept under controlled conditions (23–25 °C, 70% RH, 16:8 h light/dark photoperiod) without access to food for 24 h prior to infestation. At 45 days post-inoculation, each basil plant was infested with 20 adults (mixed instars), gently placed on the adaxial leaf surface with a fine brush; plants were enclosed in entomological cages and aphid position was periodically monitored to ensure active feeding and retention on the plant throughout the 30 h infestation period, which falls within the window in which early responses to phloem-feeding insects are typically activated [89]. After which aphids were removed. Forty-eight hours after the onset of infestation, plant tissues were harvested, weighed, immediately frozen in liquid nitrogen, and stored at −80 °C until biochemical analyses (total phenolic compounds, essential oils, and endogenous phytohormones) were performed. The experimental treatments included:

(a) non-inoculated, non-infested control plants (CONTROL:APHID−),
(b) non-inoculated plants infested with *A. pisum* (CONTROL:APHID+),
(c) PGPR-inoculated, non-infested plants (PGPR:APHID−), and
(d) PGPR-inoculated plants infested with *A. pisum* (PGPR:APHID+).

### 4.4. Essential Oil (EO) Extraction

The previously weighed plant material intended for analysis was placed in a flask containing 50 mL of distilled water. The EO was extracted by water vapor distillation using a modified Clevenger apparatus for approximately 30 min. Following distillation, the obtained distillate was supplemented with the internal standard, p-cymene (2 μL in 400 μL of methylene chloride). P-cymene was selected as the internal standard because it is not a constituent of the EO in the plant under study and does not share a similar retention time with the major compounds being analyzed.

The distillation material was then partitioned with 10 mL of methylene chloride, and the solvent was subsequently evaporated under reduced pressure at 40 °C. Next, the resulting material was injected into a gas chromatograph (GC), specifically the Trace 1300 by Thermo Fisher Scientific, Waltham, MA, USA, equipped with a TG-capillary column 5MS (30 m × 0.25 mm, 0.25 µm) following the method outlined by Banchio et al. [18]. This GC analysis enabled the quantitative determination of the monoterpenes present in the EO obtained from the different treatments. A minimum of 20 replicates were performed for each treatment. The operational conditions of the GC were as follows: the oven temperature was programmed from 60 °C (maintained for 5 min) to 240 °C at 20 °C/min, with the injector and detector set to 250 °C. A Flame Ionization Detector (FID) was used, with nitrogen as the carrier gas at a constant flow rate of 100 mL/min.

Quantification of EO constituents was performed using the internal standard p-cymene, and relative response factors were calculated according to the validated method described by Banchio et al. [18]. Compound identity was verified by matching retention times and mass spectral profiles with those reported previously for basil EO constituents.

### 4.5. Collection of Plant Volatile Organic Compounds (VOCs)

The VOC collection system comprised a vacuum pump generating a continuous airflow of 300 mL/min, and this flow rate was standardized across all samples by adjusting and verifying it with a calibrated flowmeter prior to each collection to ensure stable and identical airflow conditions. This airflow was directed through a 1000 mL polyethylene terephthalate chamber, with each chamber housing a plant according to its respective treatment. The chamber was closed at one end with a cap pre-drilled to fit the collection trap. At the other end, a cap with a hole through which the plant stem passed separated the bottom of the chamber from the base of the pot. Headspace VOCs were collected over a period of 2 h using 30 mg of Super-Q absorbent (80–100 mesh; Alltech, Deerfield, IL, USA), which was rinsed 5× with 10 mL dichloromethane prior to each collection to remove impurities. An internal standard, p-cymene (1 µg in 45 µL of methylene chloride), was added to facilitate subsequent analysis. The collected material was then injected into a GC for analysis following the method outlined by Cappellari et al. [25]. After VOC collection, each plant was dissected and weighed. VOCs were also collected from uninoculated control plants, and collections from an empty chamber confirmed that background monoterpene levels were negligible. At least ten plants were used per treatment.

### 4.6. Determination of Total Phenolic Content (TPC)

Total phenols were determined using the Folin–Ciocalteu reagent [90]. Each plant extract (0.5 mL) or gallic acid (standard phenolic compound) was mixed with Folin–Ciocalteu reagent (0.5 mL, diluted with 8 mL distilled water) and aqueous Na_2_CO_3_ (1 mL, 20% *w*/*v*). After 1 h, the level of total phenols was determined by colorimetry at a wavelength of 760 nm. Total phenol values were expressed in terms of mg gallic acid (a common reference compound) equivalent per g plant dry weight [25].

### 4.7. Determination of Phenylalanine Ammonia-Lyase (PAL) Enzyme Activity

PAL was extracted from 100 mg leaves; plant material was homogenized with liquid nitrogen using a mortar and pestle containing appropriate buffer solution (50 mM potassium phosphate and 1 mM EDTA, pH 7.8) and 1% PVP (polyvinylpyrrolidone) and then filtered through a 0.20 mm nylon filter into a centrifuge tube. The extract was centrifuged at 10,000 rpm for 20 min at 4 °C, and the supernatant was used for the enzymatic assay. Protein concentration was quantified using the Bradford method [91]. PAL activity was normalized to total soluble protein content and expressed as nmol trans-cinnamic acid produced per minute per mg protein. A heat-denatured (boiled) extract was included as a negative control to confirm the absence of non-enzymatic background activity.

### 4.8. Total RNA Extraction and Quantitative Real-Time PCR

RNA extraction from basil plants inoculated with rhizobacteria was performed according to the method described by Wang et al. [92]. Starting with 60 mg of freeze-dried plant material, the innuPREP Plant RNA Kit (Analytik Jena, Jena, Germany) was used for RNA extraction. A slight modification suggested by Kalinowska et al. [93] was incorporated: the samples were ground to a fine powder in a mortar using liquid nitrogen. The extracted RNA was verified and quantified using a NanoDrop 2000c spectrophotometer (Thermo Scientific, Waltham, MA, USA). To degrade any remaining total DNA, an additional step involving DNAase incubation was included in the provided protocol. The resulting RNA was stored at −80 °C until further use. Reverse Transcription Polymerase Chain Reaction (RT-PCR) was used to synthesize complementary DNA (cDNA) from the obtained RNA samples. The Superscript III kit (Invitrogen) was used to generate cDNA templates from 1 µg of total RNA. PCR is a molecular biology technique used to amplify a large number of copies of a DNA fragment. The experiment was conducted in a thermocycler, with a precise program. The RNA, oligonucleotides, and dNTPs were incubated together for five minutes at a temperature of 65 °C. The enzymes were added next, along with 5× First-Strand Buffer and 0.1 M DTT. The mixture was then incubated for one hour at 55 °C. Finally, the reaction was inactivated by heating it to 70 °C for 15 min. The resulting cDNA was then diluted in 80 μL of PCR-grade water and stored at −20 °C for later use.

Quantitative real-time PCR (qPCR) was performed using EvaGreen^®^ No Rox qPCR Mix (Bio&Sell, Feucht, Germany) to assess the expression of genes related to essential oil biosynthesis: *EGS* (Eugenol Synthase) and *C4H* (Cinnamate 4-Hydroxylase). Actin was used as the endogenous reference gene. Primer sequences were designed according to Rezaie et al. [94] (Table 6) and are also provided in Supplementary Table S2, including NCBI accession numbers. All samples were run in triplicate on 96-well plates. The reaction mixture contained 2 μL cDNA, 0.5 μL of each primer (final μM concentration), and 5 μL PCR-grade water in a final volume of 10 μL. Reactions were performed on a CFX Connect^TM^ Real-Time System (Bio-Rad, Munich, Germany) under the following conditions: 95 °C for 3 min, followed by 50 cycles of 95 °C for 10 s and 60 °C for 20 s. A melt curve analysis was performed at the end of the run. No-template controls were included.

To ensure analytical reliability, primer amplification efficiencies were determined from a five-fold serial dilution of pooled cDNA. All primer pairs (actin, *EGS*, *C4H*) showed efficiencies between 90–110% with correlation coefficients (R^2^) > 0.99. Actin was selected as the reference gene because its stable expression in *O. basilicum* under comparable experimental conditions has been reported previously [94,95]. In addition, actin stability across treatments was confirmed by evaluating Ct variation (<0.5 Ct), ensuring reliable normalization.

Relative gene expression was calculated using the 2^−ΔΔCt^ method, following Exposito-Rodriguez et al. [96], based on three biological replicates, each analyzed with three technical replicates.

### 4.9. Hormone Extraction

Phytohormones were extracted from 50 mg homogenized material using three rounds of extraction in 80% methanol, which was acidified to pH 2.4 with hydrochloric acid. For round 1, 400 µL, for round 2, 200 µL, and, for round 3, 100 µL solvent was used. Cell extraction was performed in 1.5 mL cryo-tubes with reinforced walls (Biozyme, Oldendorf, Germany). In order to enhance cell rupture and extraction, one steel bead of 3 mm, three steel beads of 1 mm diameter, and glass beads of 0.75 to 1 mm diameter (Carl Roth GmbH, Karlsruhe, Germany) were added to each tube, and bead milling was performed for 3 × 1 min in a homogenizer (FastPrep24, MP Biomedicals, Santa Ana, CA, USA). The extraction solvent contained deuterated hormone analogs of known concentration (d6-ABA: 12.5 µg/L; 2(d2)-JA-ILE: 12.5 µg/L; d4-SA: 50 µg/L). The combined extracts were centrifuged and stored on ice until measurement on the same day [97].

### 4.10. SPE-UPLC-MS/MS Phytohormone Analysis

Phytohormones were separated using a Nucleoshell RP Biphenyl column (100 mm × 2 mm, 2.1 µm, Macherey und Nagel, Düren, Germany) with the following gradient: 0–2 min: 5% B; 13 min: 95% B; 13–15 min: 95% B; 15–18 min: 5% B. The column temperature was set to 40 °C. Solvent A consisted of 0.3 mM ammonium formate, acidified with formic acid to pH 3.0, while solvent B was acetonitrile. The autosampler temperature was maintained at 4 °C. For sample preparation, a prototype solid-phase extraction (SPE) system was used in conjunction with UPLC. Per sample, 600 µL of plant extract was injected onto a divinylbenzene micro-SPE stationary phase (30 mg, SparkHolland B.V., Emmen, The Netherlands) at a flow rate of 200 µL min^−1^. Phytohormones were retained by the simultaneous addition of excess water (3800 µL min^−1^). Transfer from the SPE cartridge to the UPLC column was performed using 120 µL of a solution containing 20% acetonitrile and 80% solvent A. The entire procedure was carried out on a prototype system composed of a CTC Combi-PAL autosampler (1 mL injection loop), an ACE 96-well plate SPE unit, a high-pressure dispenser, an SPH1299 UPLC gradient pump, an EPH30 UPLC dilution pump, and a Mitral column oven (all from Axel-Sembrau GmbH, Sprockhövel, Germany). Phytohormones were detected by mass spectrometry using a QTrap 6500 (Sciex, Framingham, MA, USA) with electrospray ionization in positive mode. Detection was performed by multiple reaction monitoring (MRM) with a 5 ms dwell time per transition. The ion source was heated to 450 °C. The curtain gas was set to 35 psi, while ion source gases GS1 and GS2 were set to 60 psi and 70 psi, respectively. The electrospray ion spray voltage was 5500 V. To ensure analytical accuracy, calibration curves were generated for each phytohormone using authentic standards analyzed in parallel with the samples. Quantification was based on internal standard–corrected peak areas, using the deuterated analogs (d6-ABA, d4-SA, d2-JA-Ile) to correct for extraction efficiency and matrix effects. All calibration curves showed excellent linearity (R^2^ > 0.99). Method validation indicated extraction recoveries between 80–95% for all hormones, and limits of detection (LOD) and quantification (LOQ) were within the range reported for UPLC-MS/MS phytohormone assays (LOD < 0.5 ng g^−1^ FW; LOQ < 1 ng g^−1^ FW) [97].

### 4.11. Trichome Density

To determine trichome density, a layer of acrylic was applied to both sides of each leaf, carefully removed, and then mounted for microscopy in a glycerol/distilled water solution (1:10) [98]. Thirty leaf blades were processed for each treatment. The density of peltate and capitate trichomes (expressed as the number per square millimeter, number/mm^2^) was assessed by examining three random microscope fields for each leaf epidermis. Histological preparations were examined using a Nikon Eclipse 50i microscope, and images were captured using a Ds-Qi1Mc camera at 100× magnification. Trichomes were counted on both adaxial and abaxial leaf surfaces, and image analysis was performed using the Micrometrics SE Premium software (version 4.5.1).

### 4.12. Feeding Choice Tests

To determine aphid feeding preference, we used a dual-choice olfactometer consisting of two glass chambers, each holding plants according to treatment, connected by a plastic tube with a central release hole [99]. Ten aphids, starved for 24 h, were released at the center while an extractor pump established an airstream carrying plant volatiles toward the insects; after 30 min, aphid distribution was recorded as the choice. Prior to experiments, we validated the apparatus: with both chambers empty, aphids showed no directional preference and remained near the release point; when a sweet basil plant was placed in one chamber and the other left empty (alternating sides between replicates), aphids preferentially moved toward the chamber with the plant. Choice assays were then conducted between non-inoculated (control) plants and plants inoculated with GB03; each replicate used different plants, aphids were not reused, the side of each glass chamber facing the release point was covered with white paper to minimize visual cues, and the left/right positions of treatments were alternated between replicates. The experiments were repeated at least ten times.

### 4.13. Statistical Analysis

Normality and homoscedasticity of the data were first checked using Shapiro–Wilk and Levene tests, respectively. Data were then analyzed using a two-way ANOVA to assess the main effects of inoculation and herbivory, as well as their interaction, followed by Fisher’s LSD post hoc test to compare multiple treatment levels. In the figures, significant differences detected by the two-way ANOVA and post hoc comparisons are indicated by letters. In addition, paired *t*-tests were used for specific pairwise comparisons between each treatment and its corresponding control. Significant differences detected by *t*-tests are shown with asterisks. For the feeding choice assay, proportional data (percentage of aphids selecting each treatment) were analyzed using a Student’s *t*-test. Prior to analysis, proportions were arcsine square-root transformed, and residual normality was verified to ensure that parametric assumptions were met. Differences between means were considered significant at *p* < 0.05. All analyses were performed using Infostat v. 2020.

## 5. Conclusions

Aphid herbivory in *O. basilicum* activated multiple defense responses, including higher EO production, increased VOC emission, and enhanced trichome density, reflecting both biochemical and structural adjustments during the initial hours of insect feeding. When plants inoculated with *B. amyloliquefaciens* GB03 were exposed to aphid herbivory, several of these responses showed additive increases, particularly EO accumulation and VOC emission. These patterns indicate that GB03 inoculation enhanced the plant’s early defensive responsiveness, without implying a synergistic or priming-type interaction.

At the hormonal level, the combined treatment resulted in elevated SA accumulation, while JA levels remained comparable to those of aphid-infested plants, pointing to a preferential enhancement of SA-associated signaling during early response phases. These hormonal changes are consistent with the metabolic adjustments detected.

Despite these biochemical and structural modifications, aphids displayed a preference for inoculated plants, likely influenced by changes in specific volatile cues, such as increased eugenol emission. This finding highlights that early metabolic shifts do not necessarily translate into reduced herbivore attraction.

Overall, *B. amyloliquefaciens* GB03 influenced several early defense-related traits in sweet basil and altered aphid host-selection behavior, underscoring the complexity of plant–microbe–insect interactions. The responses documented. Interestingly, despite these defen here correspond to early stages of defense activation and should not be interpreted as long-term induced resistance.

## Figures and Tables

**Table 1:** Concentrations (μg/g fw) of major EO components in *O. basilicum* plants inoculated with the PGPR strain *B. amyloliquefaciens* GB03 and/or subjected to herbivory by *A. pisum*. Values are means ± standard error (SE) *(n* = 10). Different letters indicate significant differences according to Fisher’s LSD test (*p* < 0.05). Asterisks denote significant differences between aphid-infested and non-infested plants within the same inoculation treatment, according to Student’s *t*-test (*p* < 0.05).

Treatment	Cineole	Linalool	Terpineol	Eugenol
Control				
−Aphid	0.46 ± 0.20 a	0.81 ± 0.14 a	0.12 ± 0.02 a	1.77 ± 0.26 a
+Aphid	0.47 ± 0.13 ab	1.55 ± 0.29 a *	0.23 ± 0.02 ab *	3.06 ± 1.18 bc *
PGPR				
−Aphid	0.62 ± 0.12 a	1.75 ± 0.25 b	0.35 ± 0.04 b	4.69 ± 0.64 b
+Aphid	1.13 ± 0.31 b	2.83 ± 0.51 b	0.64 ± 0.13 c *	7.49 ± 1.53 c *

**Table 2:** Concentrations (ng/h g fresh weight) of major VOCs emitted by *O. basilicum* plants inoculated with the PGPR strain *B. amyloliquefaciens* GB03 and/or subjected to herbivory by *A. pisum*. Values are means ± standard error (SE) (*n* = 10). Different letters indicate significant differences according to Fisher’s LSD test (*p* < 0.05). Asterisks denote significant differences between aphid-infested and non-infested plants within the same inoculation treatment, according to Student’s *t*-test (*p* < 0.05).

Treatment	Cineole	Linalool	Terpineol	Eugenol
Control				
−Aphid	0.05 ± 0.02 a	0.11 ± 0.03 a	0.06 ± 0.02 a	0.17 ± 0.05 a
+Aphid	0.09 ± 0.03 ab	0.25 ± 0.10 a	0.17 ± 0.07 ab	1.35± 0.51 b *
PGPR				
−Aphid	0.06 ± 0.01 a	0.19 ± 0.06 a	0.05 ± 0.01 a	0.32 ± 0.05 b
+Aphid	0.16 ± 0.04 b *	0.55 ± 0.12 b *	0.27 ± 0.04 b *	1.66± 0.29 c *

**Table 3:** PAL activity (μg trans-cinnamic acid min^−1^ mg protein^−1^) in *O. basilicum* plants inoculated with the PGPR strain *B. amyloliquefaciens* GB03 and/or subjected to herbivory by *A. pisum*. Values are means ± standard error (SE) (*n* = 10). Different letters indicate significant differences according to Fisher’s LSD test (*p* < 0.05). Asterisks denote significant differences between aphid-infested and non-infested plants within the same inoculation treatment, according to Student’s *t*-test (*p* < 0.05).

	PAL
Control	
−Aphid	23.11 ± 4.33 a
+Aphid	38.15 ± 3.77 ab *
PGPR	
−Aphid	42.10 ± 5.36 b
+Aphid	42.93 ± 5.37 b

**Table 5:** Density (n/mm^2^) of PT and CT on the abaxial and adaxial leaf surfaces of *O. basilicum* plants inoculated with the PGPR strain *B. amyloliquefaciens* GB03 and/or subjected to herbivory by *A. pisum*. Values are means ± standard error (SE) (*n* = 20). Different letters indicate significant differences according to Fisher’s LSD test (*p* < 0.05). Asterisks denote significant differences between aphid-infested and non-infested plants within the same inoculation treatment, according to Student’s *t*-test (*p* < 0.05).

	Abaxial Face		Adaxial Face	
	Peltate	Capitate	Peltate	Capitate
Control				
−Aphid	2.13 ± 0.15 a	4.15 ± 0.42 a	1.40 ± 0.08 a	3.77 ± 0.40 a
+Aphid	2.87 ± 0.29 b *	4.18 ± 0.27 a	1.87 ± 0.17 b *	4.43 ± 0.43 b *
PGPR				
−Aphid	2.99 ± 0.19 b	4.21 ± 0.38 a	1.77 ± 0.12 b	4.27 ± 0.35 a
+Aphid	2.78 ± 0.19 b	5.18 ± 0.50 a	1.80 ± 0.11 b	5.78 ± 0.40 b *

**Table 6:** Primer sequences for RT-PCR.

Gene	Forward Primer Sequence (5′-3′)	Reverse Primer Sequence (5′-3′)
*Actin*	GCAGGGATCCACGAGACCC	CCCACCATGAGCACCAC
*EGS*	ACCCATAGCAATCCTTCACTG	AGTTGAAGCCTCCACATCGT
*C4H*	GCCAACAACCCCGCTCAATG	CCAACGCCGAAGGGGAGGTATC

## Data Availability

The data presented in this study are available on request from the corresponding author.

## Supplementary Materials

The following supporting information can be downloaded at: https://www.mdpi.com/article/10.3390/plants15010015/s1, Table S1: Two-way ANOVA results (*F*- and *p*-values) for inoculation, aphid herbivory, and their interaction across all measured parameters of *O. basilicum*. Table S2: Accession numbers, forward and reverse primer sequences, and annealing temperatures (Ta) used for the quantitative real-time PCR analysis in *O. basilicum*.
