# Supplementary material for: Enhanced Biochemical and Structural Defense in PGPR-Inoculated Sweet Basil Under Aphid Herbivory
Source: Plants (Basel). 2025 Dec 20;15(1):15. doi: 10.3390/plants15010015 (PMC12787337; doi:10.3390/plants15010015)
Supplement: Supplementary file 1 [file plants-15-00015-s001.zip › plants-4051237-supplementary.pdf]

Supplementary Table 1. Two-way ANOVA results (F- and p-values) for inoculation, aphid herbivory, and their interaction across all measured parameters of *O. basilicum*.

	Model	Inoculation	Aphid	Interaction
EO yield	F=12.94, p=<0.0001	F=28.99, p=0.0024	F= 9.29, p=<0.0001	F=0.45, p=0.4226
cineole	F=2.44, p=0.0703	F=4.03, p=0.0479	F=1.64, p=0.2040	F=1.43, p=0.2353
linalool	F=5.88, p=0.0011	F=9.79, p=0.0025	F=6.55. p=0.0124	F=0.23, p=0.6318
terpineol	F=9.67, p=<0.0001	F=20.07, p=<0.0001	F=7.89, p=0.0066	F=1.62, p=0.2081
eugenol	F=14.64, p=0.0021	F=11.06, p=0.0068	F=29.03, p=0.0049	F=2.81, p=0.9070
Total VOC emission	F=9.91, p=0.0001	F=4.28, p=0.0483	F=18.56, p=0.0002	F=1.34, p=0.2572
VOC cineole	F=3.06, p=0.0409	F=1.76, p=0.1933	F=5.21, p=0.0286	F=1.25, p=0.2703
VOC linalool	F=3.87, p=0.0172	F=3.33, p=0.0766	F=5.89, p=0.0205	F=1.07, p=0.3084
VOC terpineol	F=3.48, p=0.0259	F=0.52, p=0.4737	F=8.63. p=0.0058	F=0.89, p=0.3507
VOC eugenol	F=5.18, p=0.0046	F=0.22, p=0.6417	F=14.76, p=0.0005	F=0.19, p=0.6684
TPC	F=3.55, p=0.0214	F=10.61, p=0.0021	F=0.0053, p=0.9817	F=0.11, p=0.7392
Pal activity	F=3.32, p=0.0307	F=5.57, p=0.0239	F=2.48, p=0.1242	F=1.99, p=0.1668
C4H	F=4.10, p=0.0491	F=11.49, p=0.0095	F=0.75, p=0.4130	F=0.06. p=0.8157
EGS	F=22.10, p=0.0003	F=41.71, p=0.0002	F=23.43, p=0.0013	F=1.16, p=0.3124
JA	F=5.83, p=0.0027	F=6.64, p=0.0148	F=1.42, p=0.2416	F=9.42, p=0.0044
JA-ile	F=3.87, p=0.0181	F=0.72. p=0.4023	F=6.21, p=0.0181	F=4.68, p=0.0381
OPDA	F=3.20, p=0.0365	F=1.33, p=0.2576	F=7.07, p=0.0121	F=1.19, p=0.2835
SA	F=17.83, p=<0.0001	F=9.98, p=0.0035	F=37.96, p=<0.0001	F=5.32, p=0.0279
ABA	F=2.58, p=0.0705	F=1.70, p=0.2013	F=0.83, p=0.3679	F=5.21, p=0.0292
PT abaxial	F=3.99, p=0.0093	F=3.52, p=0.0628	F=1.66, p=0.2005	F=5.47. p=0.0208
CT abaxial	F=1.40, p=0.2471	F=1.71, p=0.1936	F=1.50, p=0.2229	F=1.33, p=0.2515
PT adaxial	F=3.00, p=0.0333	F=1.49, p=0.2249	F=4.30, p=0.0402	F=3.13, p=0.0794
CT adaxial	F=5.24, p=0.0020	F=4.91, p=0.0285	F=6.78, p=0.0103	F=1.05, p=0.3069

Supplementary Table 2. Accession numbers, forward and reverse primer sequences, and annealing temperatures (Ta) used for the quantitative real-time PCR analysis in *O. basilicum*.

Gene	Accession number	Primer sequences	Product size (bp)
Actin	AF282624	F: GCAGGGATCCACGAGACCC<br>R: CCCACCATGAGCACCAC	95
EGS	DQ372812.1	F: ACCCATAGCAATCCTTCACTG<br>R: AGTTGAAGCCTCCACATCGT	85
C4H	HM990150.1	F: GCCAACAACCCCGCTCAATG<br>R: CCAACGCCGAAGGGGAGGTATC	119
